# Characterization of Erythroferrone in a Teleost Fish (*Dicentrarchus labrax*) With Two Functional Hepcidin Types: More Than an Erythroid Regulator

**DOI:** 10.3389/fimmu.2022.867630

**Published:** 2022-04-08

**Authors:** João V. Neves, Carolina Barroso, Pedro Carvalho, Magda Nunes, José F. M. Gonçalves, Pedro N. S. Rodrigues

**Affiliations:** ^1^ Instituto de Investigação e Inovação em Saúde (i3S), Universidade do Porto, Porto, Portugal; ^2^ Iron and Innate Immunity, Instituto de Biologia Molecular e Celular (IBMC), Universidade do Porto, Porto, Portugal; ^3^ Instituto de Ciências Biomédicas Abel Salazar (ICBAS), Universidade do Porto, Porto, Portugal; ^4^ Programa Doutoral em Biologia Molecular e Celular (MCbiology), Instituto de Ciências Biomédicas Abel Salazar (ICBAS), Universidade do Porto, Porto, Portugal

**Keywords:** erythroferrone, hepcidin, teleost fish, iron overload, anemia, infection

## Abstract

Erythroferrone is a recently identified erythroid regulator produced by erythroblasts in the mammalian bone marrow and extramedullary sites, known to be induced in conditions of anemia or blood loss. Iron metabolism is affected by erythroferrone through its capacity to inhibit hepcidin production, leading to the increase of iron availability required for erythropoiesis. However, little is known about erythroferrone function in other vertebrates, in particular teleost fish, that unlike mammals, present two different functional types of hepcidin, one type mostly involved in iron metabolism and the other in antimicrobial response. The study of erythroferrone evolution and its biological role in teleost fish can give us valuably new insights into its function. To address these questions, we characterized erythroferrone in the European sea bass (*Dicentrarchus labrax*), a species presenting two hepcidin types, and evaluated variations in its expression levels in response to different experimental conditions. During experimental anemia, erythroferrone responds by increasing its expression and suppressing hepcidin production, following the pattern observed in mammals, but it is not influenced by iron overload. However, during bacterial infection, erythroferrone is downregulated and hepcidin levels increase. Furthermore, administration of Hamp1 but not of Hamp2 peptides suppresses erythroferrone expression. In conclusion, in dual hepcidin teleost fish erythroferrone seems to only interact with type 1 hepcidin, known to be involved in iron homeostasis, but not with type 2, which has an almost exclusive antimicrobial role.

## Introduction

Iron is an essential element for most living organisms, being involved in a myriad of metabolic processes, such as oxygen transport and storage (through haemoglobin and myoglobin), cell replication, as a co-factor of many enzymes and also with a role in the immune response (1). Iron can also have serious deleterious effects, due to its ability to easily form free oxidative radicals, which in excess and if not properly neutralized, can cause damage to cells, proteins and DNA. Therefore, preserving iron homeostasis is critical to maintain a healthy state. The key player for this homeostasis is hepcidin, a small cysteine rich peptide that was originally identified as an antimicrobial peptide, but quickly became the central player in iron metabolism (2–5). Hepcidin interacts with the sole known iron exporter, ferroportin, by producing conformational changes and causing its internalization and ubiquitination in hepatocytes, macrophages and intestinal enterocytes, thus reducing iron export and mobilization (6, 7). In conditions of excess iron, hepcidin levels increase in order to limit iron absorption, whereas during iron deficiency, such as due to blood loss, anemia or hypoxia, hepcidin is supressed, allowing for iron release and mobilization, and, indirectly, for an increase in absorption (8, 9). Hepcidin also has an important role during infectious processes, by limiting iron release from the cells and thus limiting its availability for pathogen growth and proliferation. However, during prolonged infections, this also poses a risk for the host, since iron is also unavailable to maintain erythropoiesis, leading to the so called anemia of inflammation, which can increase in severity the longer the infectious processes last, and thus become fatal to the host (10–12).

Another known erythroid regulator is erythropoietin (EPO), which comes into play when there is a need for an enhanced erythropoiesis, such as during anemia or hypoxia, leading to a decrease in hepcidin to allow increased iron release and mobilization (13, 14). Till recently, the mechanisms of interaction between EPO and hepcidin were not fully understood, but that changed with the discovery of yet another novel erythroid regulator, erythroferrone (ERFE) (15, 16). ERFE was originally identified as Fam132b, encoding for a secreted protein member of the C1q/TNF-related protein family, named CTRP15 or more commonly myonectin (17). When enhanced erythropoiesis is required, there is an increase in EPO production by the kidney which leads to an increase in erythroblast production and also to an increase in the release of ERFE to the bloodstream by the erythroblasts. ERFE will then supress hepcidin production in the hepatocytes, by interfering with the BMP/SMAD signalling pathway (18), thus allowing for an increase in iron release and mobilization from hepatocytes and erythrocyte recycling macrophages, as well as an increase in dietary absorption by the intestine.

All of these players can be identified in the genome database of many teleost fish species. However, apart from the well-studied hepcidin and erythropoietin (19, 20), functional information about fish erythroferrone is almost non-existent, opening the question of its biological role in these vertebrates. In addition, the fact that many teleost fish present two different functional types of hepcidin raises the question of erythroferrone interactions and function in fish. In teleosts, type 1 hepcidin, of which usually a single copy is present, is generally more involved in iron homeostasis, in a similar way to mammalian hepcidin, whereas type 2 hepcidin, of which multiple copies can exist, present a significant role in the immune response to a variety of pathogens (21, 22).

In order to better understand the role of erythroferrone and its relation with each type of hepcidin in teleost fish, we have identified and characterized it in a fish species with two functional hepcidin types, the European sea bass (*Dicentrarchus labrax*) (21). We have evaluated the erythroferrone response in conditions of iron overload, anemia, bacterial infection, and studied the impact of each hepcidin type in its expression. With this, we hope to contribute for a better understanding of the evolution of erythroferrone and its role in erythropoiesis and iron homeostasis.

## Materials and Methods

### Animals

Healthy European sea bass (*Dicentrarchus labrax*), with an average weight of 50 g, were provided by a commercial fish farm in the north of Spain (Sonríonansa S.L., Pesués, Cantabria, Spain) (22, 23). Prior to the experiments, fish were acclimated for 30 days to the fish holding facilities of the Instituto de Ciências Biomédicas Abel Salazar (ICBAS), Porto. Fish were kept in 110 liters recirculating sea water (28 ± 1‰ salinity) tanks at 22 ± 1°C, with a 13/11-hour light/dark cycle and fed daily *ad libitum* with commercial fish feed with an iron content of approximately 200 mg iron/kg feed. Before each treatment, fish were anaesthetized with ethylene glycol monophenyl ether (2-phenoxyethanol, 3 mL/10L, Merck, Algés, Portugal). All animal experiments were carried out in strict compliance with national and international animal use ethics guidelines (including ARRIVE guidelines), approved by the animal welfare and ethic committees of ICBAS (permits P293/2019/ORBEA and P375/2020/ORBEA) and DGAV – Direcção-Geral da Alimentação e Veterinária, and conducted by experienced and trained FELASA Function A+B+D investigators.

### Isolation of Sea Bass Erythroferrone Gene

Pairs of oligonucleotide PCR primers were designed according to conserved regions of *erfe* mRNA sequences from other fish and mammalian species, available in the National Center for Biotechnology Information nucleotide database (http://www.ncbi.nlm.nih.gov/) and Ensembl (http://www.ensembl.org/) and cDNA preparations from spleen and kidney were used in PCR amplifications (22, 23). PCR products were run on 1.2% agarose gels, relevant fragments purified with the NZYGelpure kit (NZYtech, Lisbon, Portugal), cloned into pCR™2.1-TOPO^®^ vectors, propagated in One Shot^®^ Mach1™-T1R competent cells (Invitrogen, Life Technologies, Carlsbad, CA) and sent for sequencing (Eurofins Genomics, Ebersberg, Germany). Both strands were sequenced, and chromatograms were analyzed in FinchTV (Geospiza, Seattle WA, USA) and assembled using Multalin (http://bioinfo.genopole-toulouse.prd.fr/multalin/multalin.html).

### Genomic Organization

Genomic DNA was isolated from sea bass red blood cells using the NZY Blood gDNA Isolation kit (NZYTech), according to the manufacturer’s instructions; quantification was performed using a NanoDrop 1000 spectrophotometer (Thermo Fisher Scientific); quality was checked by agarose gel electrophoresis (22, 23). One microgram of genomic DNA was amplified by RT-PCR with the primers based on the previously obtained cDNA sequences, with the following cycling profile: 94°C for 5 min, 30 cycles of 94°C for 90 s, 59°C for 60 s, and 72°C for 90 s. Several PCR products were purified, cloned, and sent for sequencing as described earlier. Comparisons were made between cDNA and genomic DNA to assess the similarity of the coding regions and to identify intron/exon boundaries. A comparison between the genomic sequences of sea bass erythroferrone with those of other vertebrates was made, with Ensembl accession numbers for sequences used as follows: *Homo sapiens* (ENST00000546354); *Mus musculus* (ENSMUST00000086861); *Monodelphis domestica* (ENSMODT00000020244); *Anolis carolinensis* (ENSACAT00000006555); *Ornithorhynchus anatinus* (ENSOANT00000024567); *Xenopus tropicalis* (ENSXETT00000049409); *Sparus aurata* (ENSSAUT00010007639); *Gasterosteus aculeatus* (ENSGACT00000002111); *Esox lucius* (ENSELUT00000026569); *Cyprinodon variegatus* (ENSCVAT00000011742); *Parus major* (ENSPMJT00000028878); *Serinus canaria* (ENSSCAT00000008685).

### Amplification of 5’ and 3’ Flanking Regions

The 5’ and 3’ RACE were carried out as previously described (22, 23) using the 5’/3’ RACE Kit, 2nd Generation (Roche Applied Science, Amadora, Portugal) according to the manufacturer’s instructions. Conditions for PCR were as follows: 94°C for 2 min, 94°C for 15 s, 59°C for 30 s, 72°C for 40 s, for 10 cycles; 94°C for 15 s, 59°C for 30 s, 72°C for 40 s (plus 20 s/cycle), for 25 cycles, with a final elongation at 72°C for 7 min. When necessary, a second PCR amplification was performed using the same conditions for an additional 30 cycles. Amplification products were run on agarose gels, relevant fragments purified, cloned, and sequenced as previously described.

### Sequence Alignment and Phylogenetic Analysis

Amino acid sequence alignments were performed using CLUSTALW from MEGAX (24). A phylogenetic tree was constructed using the Maximum Likelihood method, with the Jones–Taylor–Thornton (JTT) model, Nearest-Neighbor-Interchange heuristic model, complete deletion of gaps, and 10000 bootstrap replications. Sequences used for comparisons and phylogenetic trees include organisms representative of the various teleost fish and tetrapod classes, and their accession numbers were as follows: *Homo sapiens* (NP_001278761); Mus musculus (NP_775571); *Monodelphis domestica* (XP_007502443); *Monodon monoceros* (XP_029081431); *Camelus dromedaries* (KAB1279206); *Ornithorhynchus anatinus* (XP_001513652); *Corvus cornix cornix* (XP_010403787); *Taeniopygia guttata* (XP_030136162); *Parus major* (XP_015492724); *Alligator mississippiensis* (XP_014454239); *Anolis carolinensis* (XP_008104647); *Xenopus tropicalis* (NP_001072387); *Carassius auratus* (XP_026137994); *Cyprinus carpio* (KTG44855); *Danio rerio* (XP_002660750); *Electrophorus electricus* (XP_026885811); *Esox lucius* (XP_010890517); *Fundulus heteroclitus* (XP_021170161); *Gadus morhua* (XP_030219798); *Ictalurus punctatus* (XP_017351597); *Lates calcarifer* (XP_018534513); *Larimichthys crocea* (XP_019134833); *Maylandia zebra* (XP_004572440); *Oncorhynchus kisutch* (XP_020323002); *Oncorhynchus mykiss* (XP_021468332); *Oreochromis niloticus* (XP_013121495); *Oryzias latipes* (XP_004078828); *Paralichthys olivaceus* (XP_019965497); *Salmo salar* (XP_013994632); *Salmo truta* (XP_029559874); *Seriola lalandi dorsalis* (XP_023282631); *Sparus aurata* (XP_030259008)

### Molecular Modeling of Sea Bass Erythroferrone

Prediction of the three-dimensional structure of sea bass erythroferrone was performed by protein homology detection/modeling, using the Phyre2 Protein Fold Recognition Server (25). To maximize sequence coverage and confidence, intensive modeling was selected. Obtained models were submitted to the SAVES metaserver (http://services.mbi.ucla.edu/SAVES/) to check and validate protein structures. 96.2% of the residues were found to be in the most favored and allowed regions of the Ramachandran plot. Molecular graphic images were obtained using the Polyview-3D webserver (http://polyview.cchmc.org/polyview3d.html) (26).

### RNA Isolation and cDNA Synthesis

Total RNA was isolated from tissues with the NZY Total RNA Isolation kit protocol for tissue samples (NZYtech, Lisboa, Portugal) with the optional on-column DNase treatment, according to the manufacturer’s instructions (22, 23). Total RNA quantification was performed using a NanoDrop 1000 spectrophotometer (Thermo Fisher Scientific), and quality was assessed by running the samples in an Experion Automated Electrophoresis Station (Bio-Rad, Hercules, CA). For all samples, 2.5 µg of each were converted to cDNA using the NZY First-Strand cDNA Synthesis Kit (NZYTech) according to the manufacturer’s protocol.

### Constitutive Expression of Sea Bass Erythroferrone

Five healthy sea bass were sacrificed and several tissues (namely, liver, spleen, head kidney, intestine, gill, heart and brain) were collected for RNA isolation and cDNA synthesis, as previously described (22, 23). Relative levels of *erfe* mRNA were quantified by real-time PCR analysis using a CFX384 Touch Real-Time PCR Detection System (Bio-Rad). A total of 1 µL of each cDNA sample was added to a reaction mix containing 7.5 µL iTaq Universal SYBR Green Supermix (Bio-Rad), 5 µL double distilled H_2_O, and 250 nM of each primer (**Supplementary Table 1**), making a total volume of 15 µL per reaction. A non-template control was included for each set of primers. The cycling profile was the following: 95°C for 3.5 min, 40 cycles of 95°C for 20 s and 59°C for 20 s. Samples were prepared in duplicates, a melting curve was generated for every PCR product to confirm the specificity of the assays, and a dilution series was prepared to check the efficiency of the reactions. Beta actin (*actb*) was used as the housekeeping gene. The comparative CT method (2^-ΔΔCT^ method) based on cycle threshold values was used to analyze gene expression levels.

### Gene Expression in *In Vivo* Experimental Models

*Anemia -* To induce experimental anemias, fish were individually weighted and phlebotomized from the caudal vessels the equivalent v/w of or 2% body mass (21, 22, 27), while control fish were subjected to the same manipulation (anesthesia, weighting, pinching), but no blood was removed (for a total of 80 animals randomly divided in 2 groups, 40 fish per group). *Iron overload -* To induce iron overload *(* 21, 22, 28 *)*, fish were intraperitoneally injected with 100 µl iron dextran (Sigma-Aldrich, St. Louis MO, USA) diluted in sterile PBS to a final concentration of 50 mg/mL, while control fish were injected with 100 µL sterile PBS (for a total of 80 animals randomly divided in 2 groups, 40 fish per group). *Infection* – Fish were intraperitoneally injected with 10^5^ CFU of *Photobacterium damselae* spp.*piscicida*, strain PP3, diluted in 100 µL of sterile PBS (for a total of 100 animals randomly divided in 2 groups, 50 fish per group). Control fish were similarly injected with 100 μL of sterile PBS. *Hepcidin administration* - Fish were intraperitoneally injected with commercially synthesized sea bass hepcidin peptides, either with 100 µL of a 50 µM solution of Hamp1 (QSHLSLCRWCCNCCRGNKGCGFCCKF), or Hamp2 (HSSPGGCRFCCNCCPNMSGCGVCCRF) (Bachem AG, Bubendorf, Switzerland), diluted in sterile PBS. Control fish were similarly injected with 100 µL of sterile PBS (for a total of 120 animals randomly divided in 3 groups, 40 fish per group).

In iron modulation and peptide models, fish were collected at 1, 4, 7, 10, 14 and 21 days after treatment. In the infection model, fish were collected at 1, 2, 3, 4 and 7 days post infection. Five fish from each of the experimental groups were euthanized with an overdose of anesthetic, blood collected for hematocrit determination, dissected, their tissues excised, snap frozen in liquid nitrogen and stored at -80°C until further use. RNA was isolated and converted into cDNA, and gene expression analysis (*erfe*, *epo*, *hamp1*, *hamp2* and *hbb*) was conducted as previously described for the liver, spleen and head kidney. No animals were excluded in any of the experiments.

### Statistical Analysis

Statistical analysis was carried out using GraphPad Prism 8 (GraphPad Software Inc, La Jolla CA, USA). The normality of the distribution was evaluated with the Kolmogorov-Smirnov test. Multiple comparisons were performed with One-way ANOVA and *post hoc* Student Newman-Keuls test. A p value <0.05 was considered statistically significant.

## Results

### Molecular Characterization of Sea Bass Erythroferrone

A single sea bass erythroferrone transcript was obtained by PCR amplification and 5’/3’ RACE. Erythroferrone coding DNA (deposited in GenBank under accession number MT043300) consists of an open reading frame of 918 bp, with flanking 5’ and 3’ untranslated regions of 356 bp and 2090 bp, respectively, and encodes a 305 amino acid protein. The peptide has a predicted MW of 32870 Da and an isoelectric point of 5.29. A signal peptide cleavage site was predicted between positions 36 and 37 (MEA-AS) with the SignalP-5.0 server (http://www.cbs.dtu.dk/services/SignalP/), and several protein features were identified using Prosite (https://prosite.expasy.org/scanprosite/): a complement component 1q (C1q) domain profile (between positions 152-305), a proline rich region (105-125) and three N-glycosylation sites (197-200, 246-249 and 284-287) (**Supplementary Figure 1**).

**Figure 1:**
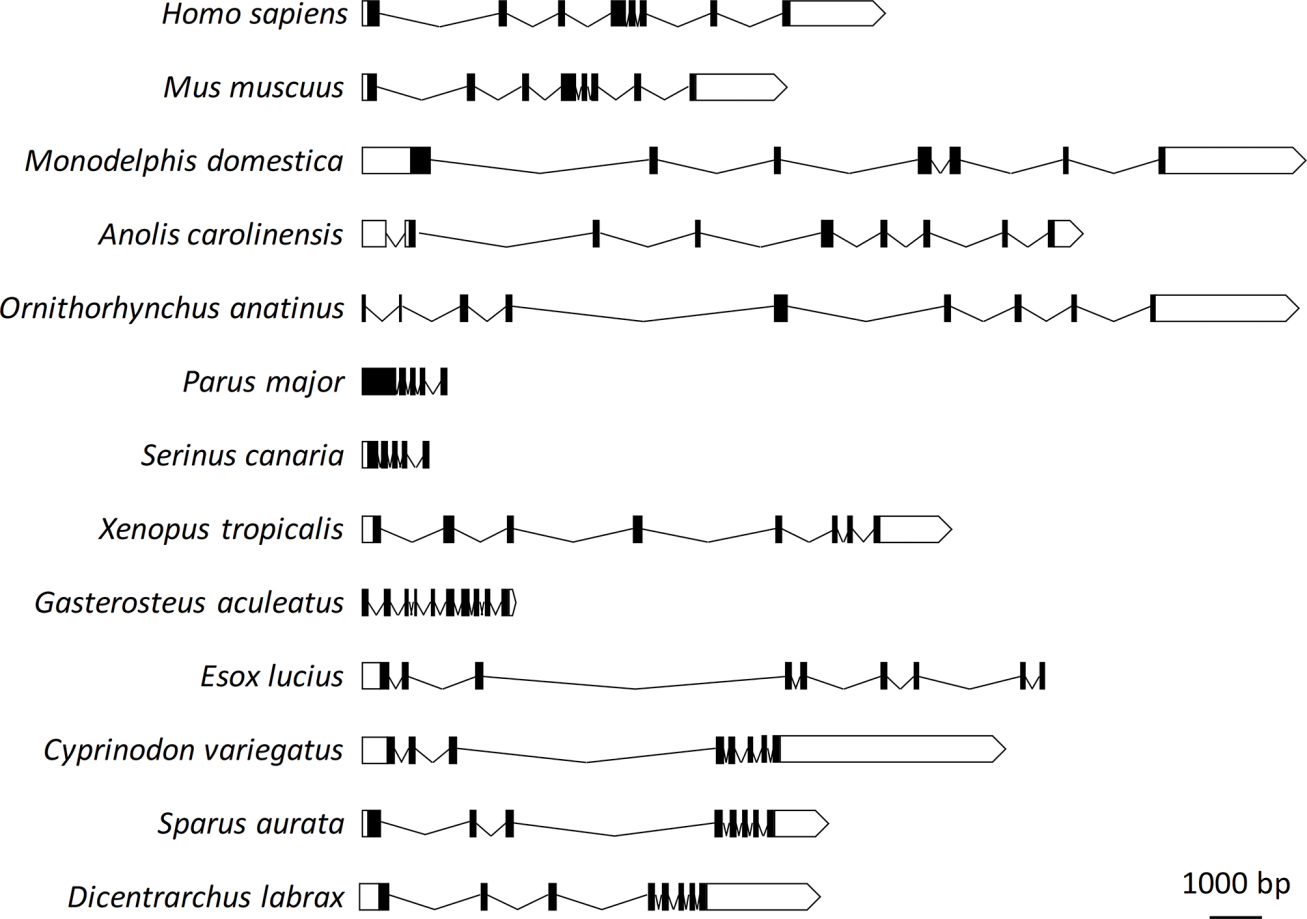
Genomic organization of sea bass erythroferrone and comparative view with other vertebrate species. Exons are shown as black boxes, introns as solid lines, and untranslated regions as white boxes.

The genomic structure of sea bass erythroferrone consists of eight exons and seven introns, with a single initiator methionine and stop codon. The genomic interval from the initiator methionine to the stop codon is 6952 bp. Genomic organization of sea bass erythroferrone was analyzed and compared with those of other vertebrates, including mammals, amphibians, birds, reptiles and other fish. Significant variations were observed in terms of exon/intron numbers, as well as sizes, not only between vertebrate classes, but also between different fish species (**Figure 1**).

### Sequence Comparison and Phylogenetic Analysis

**Figure 2:**
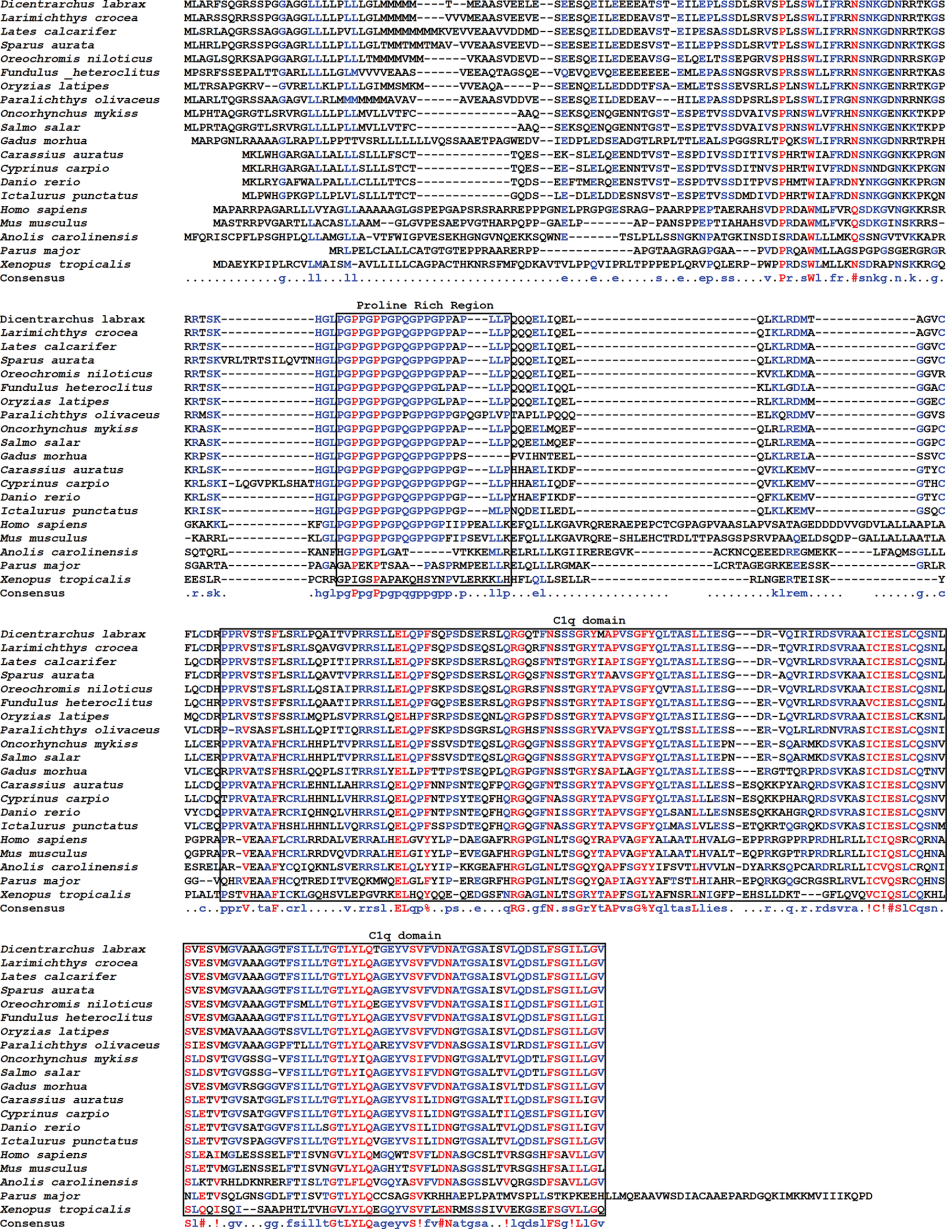
Sequence alignment of erythroferrone proteins. Sea bass erythroferrone protein was aligned with erythroferrones from other vertebrates. Gaps in the alignment are indicated by dashes, and colours represent different degrees of conservation: red, high; blue, moderate conserved; black, low. Consensus sequence is presented below the alignment.

Sequence comparison with other vertebrate species erythroferrone proteins showed a high degree of conservation particularly among fish species, with sequence identity varying between 57.48% (*D. rerio*) and 93.46% (*L. crocea*), while the lowest identity observed was with birds, of 26.47% (*P. major*) (**Supplementary Table 2**). The highest degree of sequence conservation is seen at two of the conserved features, the proline rich region and the C1q domain profile (**Figure 2**).

Additionally, although we can observe some conservation of the N-terminus among the various fish species, this region is less conserved when compared with tetrapods.

**Figure 3:**
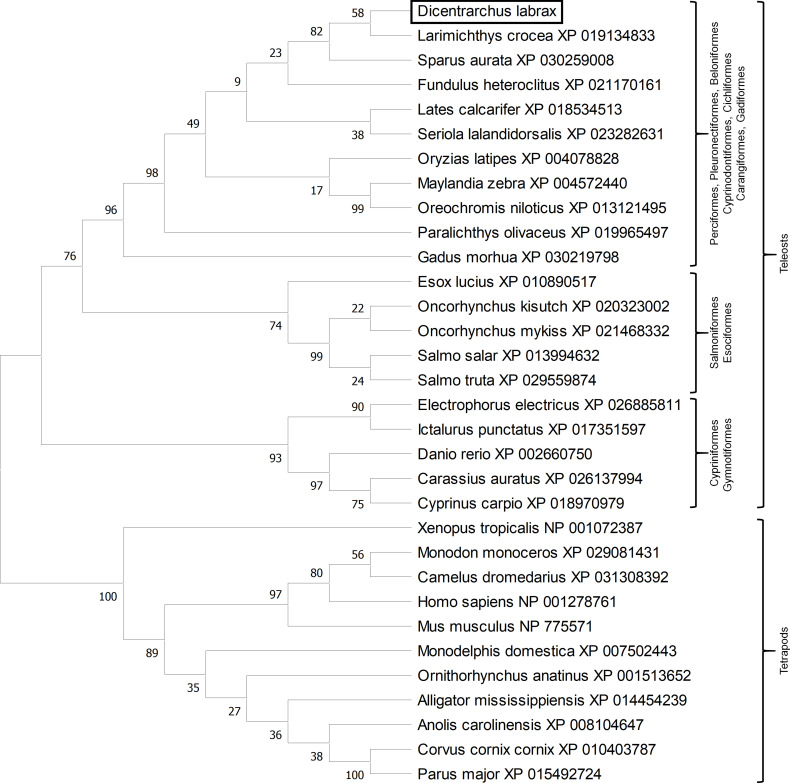
Evolutionary analysis by Maximum Likelihood method. The evolutionary history was inferred by using the Maximum Likelihood method and JTT matrix-based model. The bootstrap consensus tree inferred from 10000 replicates is taken to represent the evolutionary history of the taxa analysed. The percentage of replicate trees in which the associated taxa clustered together in the bootstrap test (10000 replicates) are shown next to the branches. Initial tree(s) for the heuristic search were obtained automatically by applying Neighbor-Join and BioNJ algorithms to a matrix of pairwise distances estimated using a JTT model, and then selecting the topology with superior log likelihood value. This analysis involved 32 amino acid sequences. All positions with less than 95% site coverage were eliminated, i.e., fewer than 5% alignment gaps, missing data, and ambiguous bases were allowed at any position (complete deletion option). There were a total of 173 positions in the final dataset. Evolutionary analyses were conducted in MEGA X.

Phylogenetic analysis clusters fish erythroferrones separated from tetrapods (**Figure 3**) and among fish, there seem to be three distinct clusters. One cluster includes members of the Cypriniformes and Gymnotiformes orders, a second cluster includes Salmoniformes and Esociformes and a third and more heterogeneous cluster, where sea bass erythroferrone is located, includes fish from the Perciformes, Pleuronectiformes, Beloniformes, Cyprinodontiformes, Cichliformes, Carangiformes and Gadiformes orders.

### Molecular Modeling of Erythroferrone C1q Domain

**Figure 4:**
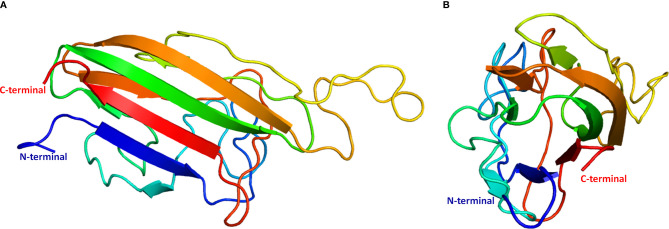
Theoretical model of sea bass erythroferrone C1q domain 3D structure. **(A)** side view; **(B)** top view. Ribbons are rainbow coloured from blue to red, starting from the N-terminus and ending in the C-terminus.

To predict protein structure, a profile-profile alignment webserver (Phyre2) was used to search for sequence similarities between sea bass erythroferrone and other proteins with known 3D structures that could serve as template(s) for homology modeling. Only the region corresponding to the C1q domain (comprised by amino acids 152-305) was successfully modelled, as of yet there are no available templates for a full length erythroferrone protein. Phyre2 selected 6 templates with a confidence level of 100% to model the protein: (1) globular head of the complement system protein C1q, PDB d1pk6c, 19% identity; (2) single chain recombinant globular head of the complement system protein C1q, PDB c5hkjA, 19% identity; (3) structure of the human collagen x nc1 trimer, PDB c1gr3A, 19% identity; (4) structure of the human collagen x nc1 trimer, PDB d1gr3a, 19% identity; (5) crystal structure of a single-chain trimer of human adiponectin globular domain, PDB c4douA, 18% identity; (6) globular domain of zebrafish complement 1qA protein, PDB c5hbaA, 11% identity. From a total of 154 residues, 136 (88%) were modeled at 100% confidence, whereas the remaining 18 (12%) were modeled *ab initio*. Secondary structure predictions reported by Phyre2 indicate a beta sheet configuration, with several disordered areas connecting the various beta strands (**Figure 4**).

### Sea Bass Erythroferrone Is Highly Expressed in Hematopoietic Organs

**Figure 5:**
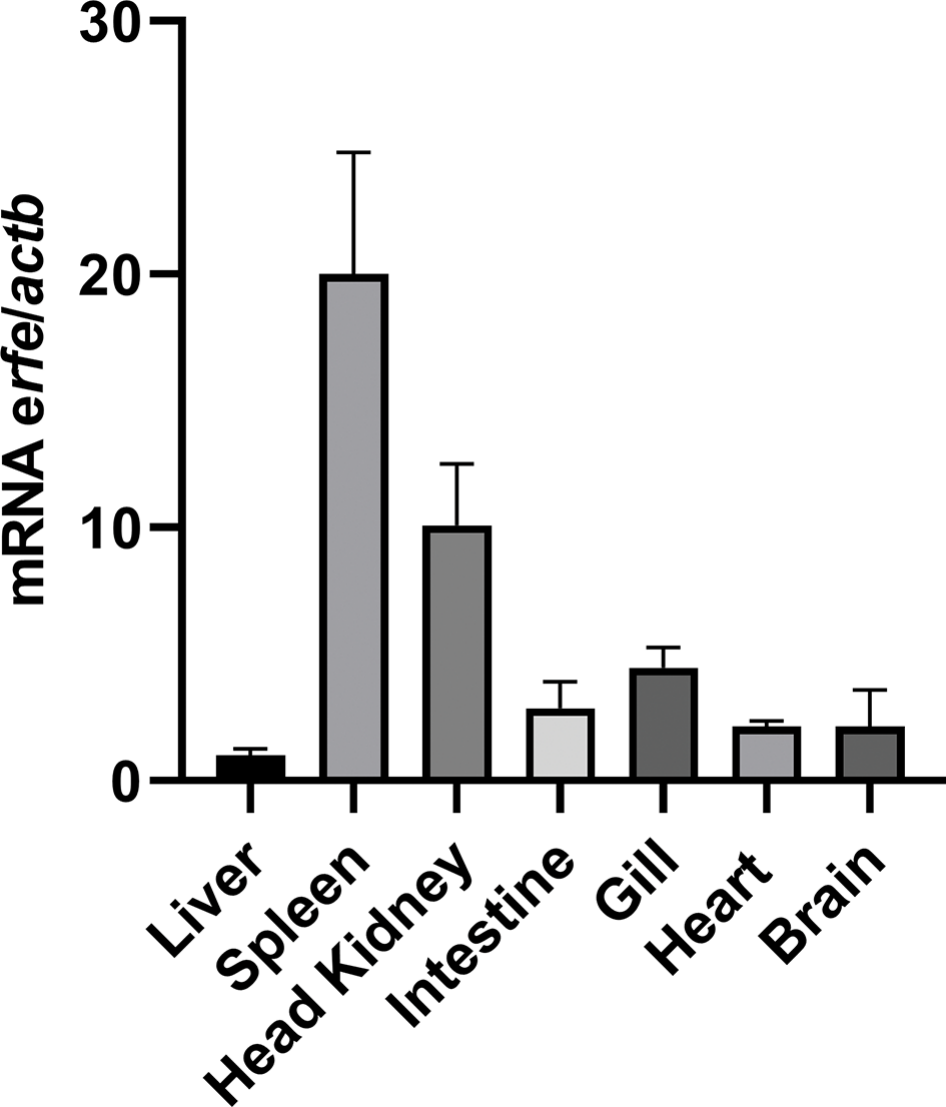
Constitutive expression of erythroferrone in several sea bass tissues. Expression was measured by real-time PCR in several organs of healthy sea bass specimens. Values are expressed as means ± SD (n=5). *Actb* was used as the housekeeping gene.

Constitutive expression of sea bass erythroferrone was evaluated in several healthy sea bass tissues, namely, liver, spleen, head kidney, intestine, gill, heart and brain (**Figure 5**). Highest expression was observed in the spleen, followed by the head kidney, which is not surprising, considering these are the major hematopoietic organs in teleost fish. Moderate expression was observed in the intestine, gill, heart and brain, with the liver presenting the lowest constitutive expression of all tested organs.

### Erythroferrone Expression Mirrors *hamp1*, but Not *hamp2* Expression, During Anemia

**Figure 6:**
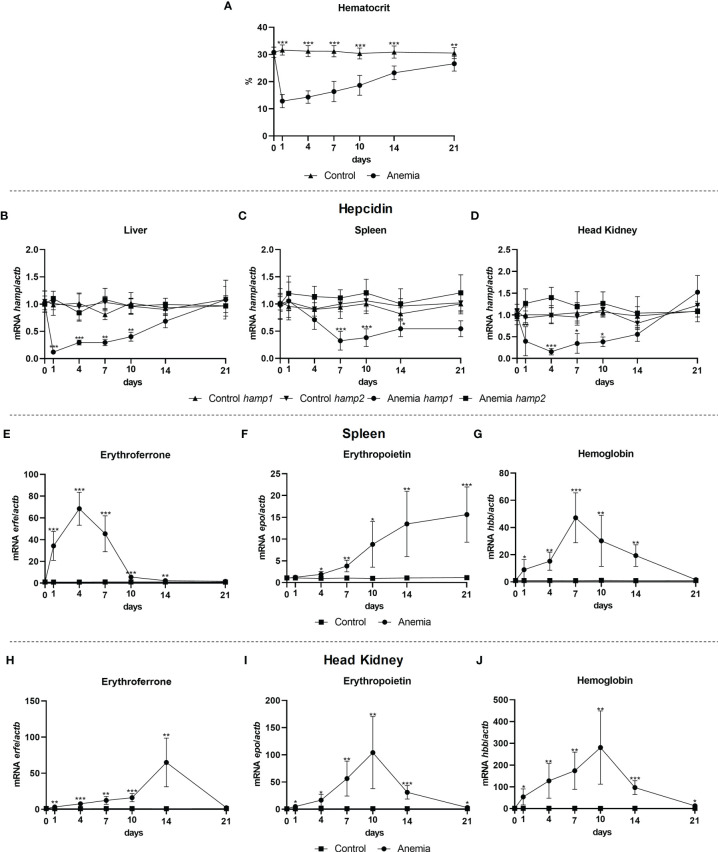
Hematocrit and gene expression in the liver, spleen and head kidney at 1, 4, 7, 10, 14, and 21 days of anemia. Variations in hematocrit levels during experimental anemia **(A)**; *Hamp1/hamp2* expression in the **(B)** liver, **(C)** spleen and **(D)** head kidney; *erfe*, *epo* and *hbb* expression in the **(E-G)** spleen and **(H-J)** head kidney. Values are expressed as means ± standard deviation (n=5). *Actb* was used as the housekeeping gene. Differences from the control group were considered significant at *P < 0.05, **P < 0.01, and ***P < 0.001.

Gene expression was evaluated in the liver, spleen and head kidney of anemic fish, at day 1, 4, 7, 10, 14, and 21 post anemia induction. As expected, a significant decrease in the hematocrit was observed as early as day 1, with a gradual recovery towards day 21 but still not completely recovering to control levels (**Figure 6A**). This anemia was accompanied by significant decreases in the expression of *hamp1* in all organs, particularly in the earlier days, with later gradual recoveries to control levels, whereas no significant variations were observed for *hamp2* (**Figures 6B-D**). Erythroferrone (*erfe*) levels in the spleen (**Figure 6E**) and head kidney (**Figure 6H**) mirrored *hamp1*, with substantial increases in expression, although reaching peak levels at different time points, with the zenith at day 4 in the spleen and 14 in the head kidney, followed in both by decreases and recovery to control levels. Increased expression of erythropoietin was also observed in the spleen (**Figure 6F**) and head kidney (**Figure 6I**), also with different patterns of expression, with a gradual increase up to day 21 in the spleen, while reaching the zenith at day 10 in the head kidney, followed by a gradual decrease to control levels. Additionally, hemoglobin levels mirrored the hematocrit, with gradual increases in both the spleen (**Figure 6G**) and head kidney (**Figure 6J**), up to day 7 and 10 respectively, followed by a return to control levels at the same time the hematocrit returned to near normal levels.

### Iron Overload Does Not Influence Erythroferrone Expression

**Figure 7:**
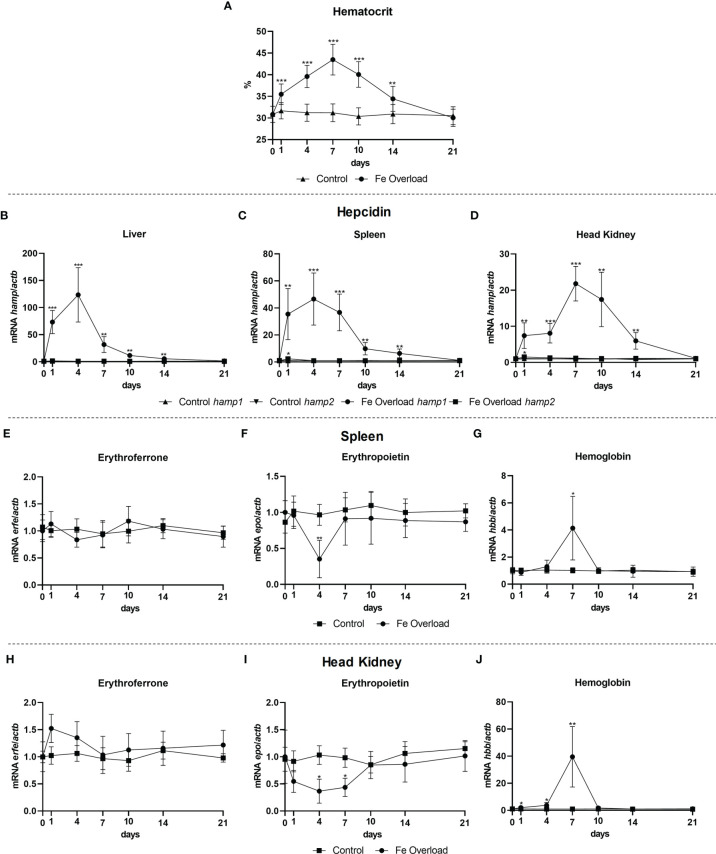
Hematocrit and gene expression in the liver, spleen and head kidney at 1, 4, 7, 10, 14, and 21 days after iron overload. Variations in hematocrit levels during experimental iron overload **(A)**; *Hamp1/hamp2* expression in the **(B)** liver, **(C)** spleen and **(D)** head kidney; *erfe*, *epo* and *hbb* expression in the **(E-G)** spleen and **(H-J)** head kidney. Values are expressed as means ± standard deviation (n=5). *Actb* was used as the housekeeping gene. Differences from the control group were considered significant at *P < 0.05, **P < 0.01, and ***P < 0.001.

Gene expression was evaluated in the liver, spleen and head kidney of iron overloaded fish, at day 1, 4, 7, 10, 14, and 21 post iron overload. A steady increase in the hematocrit was observed up to day 7, followed by a gradual decrease to control levels (**Figure 7A**). Considerable increases in expression were observed for *hamp1* in all tested tissues (**Figures 7B-D**), with similar patterns of early increase in the liver and spleen (both peaking at day 4), but a bit later in the head kidney (with the zenith at day 7), but nevertheless with recoveries to control levels in all organs at day 21 post-overload. For *hamp2*, no significant variations were observed. No significant variations in *erfe* expression were observed in the spleen (**Figure 7E**) or head kidney (**Figure 7H**), despite the slight decreases observed in erythropoietin expression in the spleen, at day 4 (**Figure 7F**), and in head kidney, at days 4 and 7 (**Figure 7I**). Furthermore, significant increases in the expression of hemoglobin were observed both in the spleen (**Figure 7G**) and head kidney (**Figure 7J**) at day 7, with a return to normal levels at day 10.

### Erythroferrone May Contribute to Hepcidin Mediated Anemia of Inflammation

**Figure 8:**
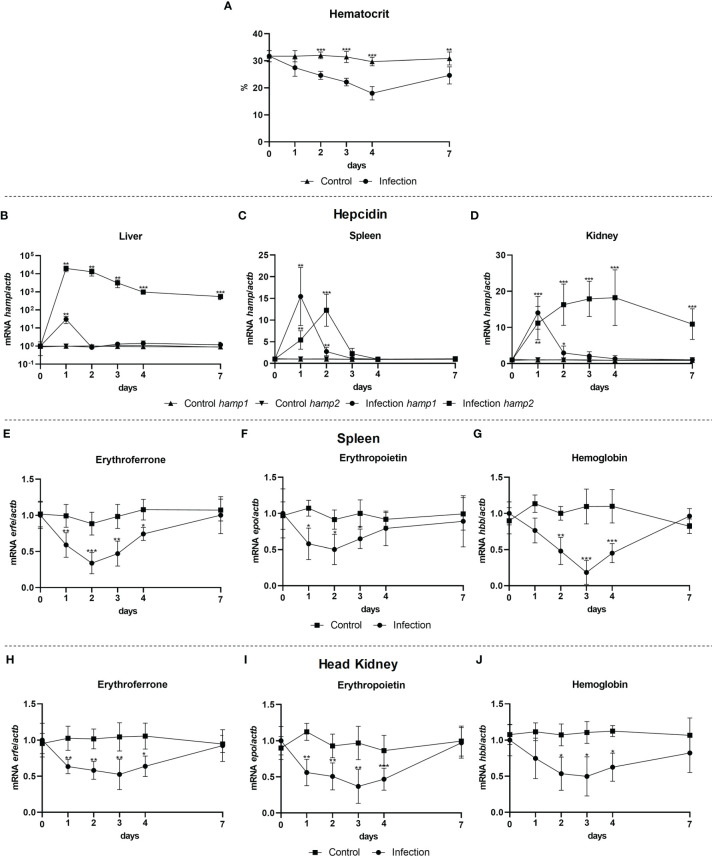
Hematocrit and gene expression in the liver, spleen and head kidney at 1, 2, 3, 4, and 7 days after bacterial infection. Variations in hematocrit levels during experimental infection with *P. damselae* spp. *piscicida* PP3 **(A)**; *Hamp1/hamp2* expression in the **(B)** liver, **(C)** spleen and **(D)** head kidney; *erfe*, *epo* and *hbb* expression in the **(E-G)** spleen and **(H-J)** head kidney. Values are expressed as means ± standard deviation (n=5). *Actb* was used as the housekeeping gene. Differences from the control group were considered significant at *P < 0.05, **P < 0.01, and ***P < 0.001.

To evaluate a possible involvement of erythroferrone during infection, gene expression was evaluated in the liver, spleen and head kidney of fish infected with the Gram-negative bacterium *P. damselae* spp. *piscicida*, 1, 2, 3, 4 and 7 post-infection. Infection had a significant impact on hematocrit, with a gradual decrease up to 4 days, followed by a recovery but still not returning to normal levels after 7 days (**Figure 8A**). Early increases in *hamp1* were observed in all tested tissues, followed by a rapid decline towards normal levels. *Hamp2* expression on the other hand was highly increased in the liver, up to 7 days, with a similar but more moderate pattern of overexpression in the head kidney, as well as a slight increase in the spleen, at 4 days post-infection (**Figures 8B-D**). Similar patterns of downregulation were observed for *erfe*, *epo* and *hbb*, in both spleen (**Figures 8E-G**) and head kidney (**Figures 8H-J**), with levels kept under normal levels mostly between days 1 and 4, and with a recovery of all genes to normal levels of expression at day 7.

### Administration of Hamp1 Strongly Reduces Erythroferrone Expression

**Figure 9:**
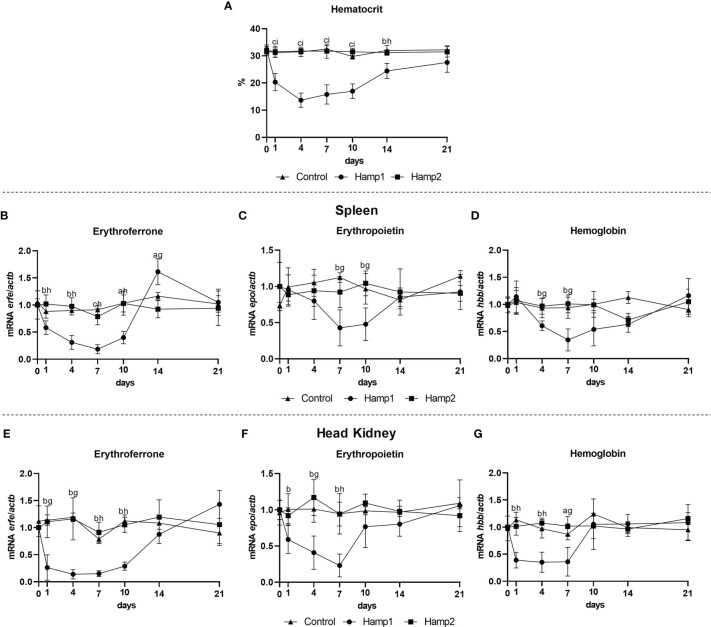
Hematocrit and gene expression in the spleen and head kidney at 1, 4, 7, 10, 14, and 21 days after intraperitoneal administration of Hamp1 or Hamp2 synthetic peptides. Variations in hematocrit levels during experimental peptide administration **(A)**; *erfe*, *epo*, and *hbb* expression in the **(B–D)** spleen and **(E–G)** head kidney after administration of Hamp1 or Hamp2 peptides. Values are expressed as means ± standard deviation (n=5). *Actb* was used as the housekeeping gene. Differences among groups were considered significant at *P*<0.05, P<0.01, and *P*<0.001, represented respectively by the letters a, b, c between control and Hamp1 administered animals, d, e, f between control and Hamp2 administered animals, and g, h, i between peptide administered groups (Hamp1/Hamp2).

It is known that erythroferrone has an effect on hepcidin, but we also wanted to know if hepcidin could have an impact on erythroferrone. As such, erythroferrone, erythropoietin and hemoglobin expressions were evaluated in the spleen and head kidney of fish administered Hamp1 and Hamp2 synthetic peptides, at day 1, 4, 7, 10, 14, and 21 post-administration. Administration of Hamp1 had a significant impact on the hematocrit, leading to a reduction starting as early as day 1, reaching the nadir at day 4 and then gradually recovering to control levels up to day 21 (**Figure 9A**); Hamp2, on the other hand, had no impact on hematocrit levels. *Erfe* expression was only influenced by the administration of Hamp1, with Hamp2 having no significant effects. Both the spleen (**Figure 9B**) and kidney (**Figure 9E**) presented similar patterns of under expression up to day 10 post-administration, but whereas in the kidney expression levels slowly recovered to control levels, in the spleen they were surpassed at day 14, with a significant overexpression followed by recovery to control levels at day 21. Similarly, *epo* and *hbb* expressions were also only influenced by Hamp1 administration, with both genes being progressively downregulated in the spleen (**Figures 9C, D**) and head kidney (**Figures 9F, G**) up to day 7, followed by gradual recoveries to normal levels.

## Discussion

Erythroferrone is a recently identified erythroid regulator, produced by erythroblasts in the bone marrow and extramedullary sites (15, 29). Erythroferrone expression increases in response to the increased levels of erythropoietin that occur in conditions of anemia or blood loss, inhibiting hepcidin production by the liver, most likely by interfering with the BMP/SMAD pathway (18, 30), and consequently inducing an increased release of iron by the hepatocytes, recycling macrophages and intestinal enterocytes, through the sole known iron exporter and target for hepcidin, ferroportin. Indirectly, it will also lead to an increase in iron absorption by the intestinal enterocytes. This iron is then used in the synthesis of hemoglobin and incorporated in novel erythroblasts, allowing for the recovery of erythrocyte levels. Information on fish erythroferrone is scarce and may show functional peculiarities since this class of animals present relevant biological differences when compared with mammals. For instance, in teleost fish, the head kidney replaces the bone marrow as the major erythropoietic organ, with the spleen also having a significant contribution in many fish species (31). Despite this, the various erythropoietic processes present many similarities to those occurring in higher vertebrate species, and involve many of the same precursors and genes. However, one crucial difference exists, which is the fact that many teleost species present not one but two different hepcidin types: type 1 hepcidin (Hamp1), usually present as a single copy, highly similar to mammalian hepcidin, and with an active involvement in iron homeostasis; and type 2 hepcidin (Hamp2), present in single or multiple copies, more dissimilar to mammalian hepcidin, and mostly involved in the host antimicrobial response (21). As such, it is likely that teleosts present a similar interaction between erythroferrone and type 1 hepcidin, but it raises questions regarding a possible involvement with type 2 hepcidins.

In order to investigate this, here we identify and characterize erythroferrone in the European sea bass (*Dicentracrhus labrax*), a teleost fish species with two hepcidin types (21). We found a single transcript, encoding for a putative 305 amino acids protein and a genome sequence that shares many similarities with its mammalian counterpart. However, when compared with erythroferrone from other vertebrate species, significant variations can be observed not only in exon/intron numbers, but also lengths, which is also reflected in protein sizes. Sequence alignment of the putative protein with erythroferrone proteins from other vertebrates shows that sea bass erythroferrone shares several of its characteristic features, namely a proline rich region and a C1q domain, which are also the regions with the highest degree of conservation among species. In fact, three-dimensional molecular modeling of sea bass erythroferrone was only possible for the C1q domain, having as templates the 3D structures of other C1q proteins, since no suitable vertebrate structures could be found for the whole erythroferrone protein. Although their exact functions are still unclear, these motifs may support multimerization (32, 33), and thus increase binding affinity of these ligands to their receptors. Sea bass erythroferrone also shares two cysteine residues at highly conserved positions (positions 238 and 243), which are present in all vertebrates, as well as two cysteines that can only be observed in fish (positions 146 and 149). The relevance of these two additional cysteines is still unknown, but they could lead to the formation of extra disulfide bonds and consequently, to alternative secondary or tertiary protein structures. Furthermore, this raises questions regarding the functionality of the N-terminal region, which although relatively conserved among fish species, is much less conserved when compared with mammalian species. In mammals this is the primary region relevant for BMP binding/interaction (34), but that remains to be confirmed in fish. Phylogenetic analysis of vertebrates erythroferrone shows two different clusters, separating fish from tetrapods and among fish, erythroferrones can be further divided in three very closely related groups, each with evolutionarily related species.

Erythroferrone mRNA was detected in all tested tissues, with the highest constitutive expressions in the head kidney and the spleen, followed my moderate expressions in the intestine, gill, heart and brain, and with the lowest expression observed in the liver. This is consistent with the functions of the kidney and spleen in teleost fish, as major erythropoietic organs (31), whereas in tetrapods that function is taken mostly by the bone marrow (29).

To better understand the role of erythroferrone in the erythropoietic processes of a fish with two functional hepcidin types, its expression was evaluated in several experimental models, which included *in vivo* models of anemia, iron overload, bacterial infection and Hamp1/Hamp2 peptide administration.

The significant decrease in hematocrit levels caused by the experimental anemia prompts the need for a compensatory erythropoiesis to replenish erythrocyte levels, leading to the modulation of hepcidin expression and consequent mobilization of iron to the erythropoietic organs, in agreement to our previous observations (27). Thus, a significant decrease in *hamp1* was observed, inducing an increase in ferroportin membrane expression, and consequent increase in iron release from hepatocytes, recycling macrophages and intestinal enterocytes, leading to its mobilization mediated by transferrin to the erythropoietic organs, to be incorporated in the hemoglobin required for the formation of new erythrocytes. We had previously shown that this suppression of *hamp1* can occur through several signaling pathways, such as the HJV/BMP/SMAD, Tf/TfR1 or the EPO/EPOR pathways (27). During conditions of anemia, it has been documented that there is an increase in the expression and release of erythropoietin, leading to a repression of hepcidin expression in the liver, likely by mechanisms such as EPOR-mediated regulation of the transcription factor C/EBPα (13), or indirectly through the suppression of SMAD4 and STAT3 signaling (35). However, some questions remain as to how exactly was this suppression mediated still persisted, and were partially answered with the discovery of erythroferrone. It has been demonstrated that increased EPO expression leads to an increase in erythroferrone (15, 36), which in turn interferes with the BMP/SMAD pathway (18, 30), leading to a suppression of hepcidin expression, and thus allowing for an increase in iron absorption, release and mobilization. This study shows that during anemia a similar mechanism is present in teleost fish, with increases in erythropoietin expression in the spleen and head kidney, as well as an increase in erythroferrone expression and hepcidin suppression. Interestingly, the time-course variations in *hamp1*, *erfe* and *epo* expressions do not fully match what is observed for mammals, where an increase in *erfe* expression in usually preceded by an increase in *epo* (15, 36). In the head kidney, we observed the traditional increase of *epo*, followed by an increase in *erfe*, but hepcidin suppression occurs sooner. In the spleen, there is a significant increase in *erfe* which somewhat matches the early decreases in *hamp1*, but precedes the increase of *epo*. As such, we must consider the existence of other mechanisms that may influence both *hamp1* and *erfe* levels during anemia, at least in fish, leading to this dissimilar dynamic to the one observed for mammals. A future analysis of this response at the protein level could shed some light on these interactions. Furthermore, since no significant changes were observed in the expression of *hamp2*, it is reliable to say that during anemia, in teleost fish, increased erythropoietin and erythroferrone levels only lead to the suppression of *hamp1* and have no impact on *hamp2* (which further corroborates the mainly antimicrobial role of this type of hepcidin).

In the experimental iron overload model, we observed a significant increase in the hematocrit, a response that occurs to cope with an excess of iron and avoid iron toxicity, by storing more iron or by using it in iron demanding processes, such as incorporating it in hemoglobin to produce more erythrocytes (37, 38). We also observed an increase in the expression of *hamp1* but not of *hamp2*, which is in accordance with the differential functions of these hepcidin types in teleost fish (21, 22). In this case, mirroring what happens during anemia, an increase in the expression of *hamp1* would have an inhibitory effect on ferroportin, limiting its expression and membrane presence, and thus limiting iron release from liver hepatocytes, macrophages and intestinal enterocytes, as well as decreased iron uptake by the intestinal enterocytes (39–41). As iron levels begin to stabilize and return to normal levels, so does the hematocrit and *hamp1* expression, signifying a return to homeostatic conditions, whereas *hamp2*, having a mainly antimicrobial role, does not change significantly. When looking at erythroferrone expression, no significant changes were observed either in the spleen or head kidney. This is noteworthy, as excess iron is known to be able to attenuate erythropoietin (42, 43), as we could also see in our experimental model, which could lead to a decrease in erythroferrone. Nevertheless, it seems that erythroferrone expression is not influenced by the increased iron availability, and as such, the observed increases in hemoglobin transcription levels and hematocrit are likely driven by other pathways, such as Erfe-independent BMP/SMAD regulation, the Tf/TfR pathway or direct interaction of EPO with the hepatic, splenic or nephric EPO receptor.

During the experimental infection, we observed a huge increase of *hamp2* expression that is kept elevated throughout the study, an expected result considering its known antimicrobial role (21, 22, 44). However, the increases in *hamp1* expression are much more limited, mostly to the initial stages of infection, and do not fully mirror the decreases in *epo* and especially *erfe*, which are more prolonged. Nevertheless, the early days suppression of *erfe* may contribute to the observed *hamp1* increases, leading to a limitation in iron availability essential to control the siderophore producing *P. damselae* proliferation (44), and thus to the early onset of anemia of inflammation. Later recoveries of *erfe*, as well as *epo* and *hbb*, to normal levels of expression, seem to match the recovery from anemia (45), although *hamp1* levels do not change significantly. As of this moment, information on fish erythroferrone is very scarce in particular during infection. The significance of these findings will need to be dressed in further studies, but there is no question for a role of erythroferrone during infection in fish. Furthermore, we must take into consideration that this is a response to a single gram-negative bacterium, and also consider that a limitation in iron availability might be advantageous or deleterious, depending on the pathogen. As such, when fish are exposed to other pathogens, we may see a different response, just like in mammalian models (16, 45–47).

In our last experimental model, we administered either Hamp1 or Hamp2 synthetic peptides to fish, and investigated their impact on erythroferrone expression. Administration of Hamp1 led to very significant anemia, due to its active role in iron metabolism and interaction with ferroportin (21, 22). Interestingly, our study demonstrates that Hamp1 also had an impact on erythropoietin, erythroferrone and hemoglobin expressions, both in the spleen and head kidney, with very significant decreases in the earlier days post administration, coinciding with the early development of anemia, and a later recovery and even over expression, matching the gradual recovery from anemia. Although it is known that increased erythropoietin/erythroferrone levels lead to a decrease in type 1 hepcidin production, the opposite has not been described. The fact remains that erythroferrone expression is influenced by Hamp1 in the early days post-administration, but it is also likely involved in the recovery from anemia in later days, resembling what happens during anemia of inflammation (45). Hamp2 administration, on the other hand, caused no significant changes in either hematocrit or gene expression, which was not totally unexpected (21, 22), since its active role is focused on antimicrobial response and not iron homeostasis.

In conclusion, we have identified and characterized erythroferrone in a teleost fish species presenting two hepcidin types, the European sea bass, and found that it only responds to type 1 hepcidin, which is known to be involved in iron homeostasis, but not with type 2 hepcidin, which has an almost exclusive antimicrobial role. We have shown that during conditions of anemia, erythroferrone responds by increasing its expression and suppressing type 1 hepcidin, following the pattern observed in mammals, but it seems to have no role during iron overload. Additionally, erythroferrone may contribute to type 1 hepcidin dependent anemia of inflammation, but the mechanisms by which this occurs do not seem to be the exact same as in mammals. Furthermore, administration of Hamp1 but not of Hamp2 peptides suppressed erythroferrone expression, although the exact mechanisms by which this occurs are unclear and will require further studies.

## Data Availability Statement

The datasets presented in this study can be found in online repositories. The names of the repository/repositories and accession number(s) can be found in the article/**Supplementary Material**.

## Ethics Statement

The animal study was reviewed and approved by Animal welfare and ethic committees of ICBAS (permits P293/2019/ORBEA and P375/2020/ORBEA) and DGAV – Direcção-Geral da Alimentação e Veterinária.

## Author Contributions

JN designed, conducted and supervised the experiments, analyzed data and wrote the paper. CB conducted experiments, analyzed data and wrote the paper. PR designed and supervised the experiments. PC, MN, and JG conducted experiments. JN had primary responsibility for final content. All authors read and approved the final manuscript.

## Funding

This work was funded by the structured program of R&D&I ATLANTIDA - Platform for the monitoring of the North Atlantic Ocean and tools for the sustainable exploitation of the marine resources (NORTE-01-0145-FEDER-000040), supported by the North Portugal Regional Operational Programme (NORTE2020), through the European Regional Development Fund (ERDF), and by funded by National Funds through FCT Fundação para a Ciência e a Tecnologia/Ministério da Ciência, Tecnologia e Ensino Superior, under project UIDB/04293/2020. CB is supported by Ph.D. fellowships SFRH/BD/114899/2016 and COVID/BD/151662/2021, financed by FCT.

## Conflict of Interest

The authors declare that the research was conducted in the absence of any commercial or financial relationships that could be construed as a potential conflict of interest.

## Publisher’s Note

All claims expressed in this article are solely those of the authors and do not necessarily represent those of their affiliated organizations, or those of the publisher, the editors and the reviewers. Any product that may be evaluated in this article, or claim that may be made by its manufacturer, is not guaranteed or endorsed by the publisher.
