# Supplementary material for: Characterization of Erythroferrone in a Teleost Fish (Dicentrarchus labrax) With Two Functional Hepcidin Types: More Than an Erythroid Regulator
Source: Front Immunol. 2022 Apr 8;13:867630. doi: 10.3389/fimmu.2022.867630 (PMC9024048; doi:10.3389/fimmu.2022.867630)
Supplement: Supplementary file 1 [file DataSheet_1.docx]

**Characterization of erythroferrone in a teleost fish (*Dicentrarchus labrax*) with two functional hepcidin types**

João V. Neves^1,2,3*^, Carolina Barroso^1,2,4^, Pedro Carvalho^3^, Magda Nunes^3^, José F.M. Gonçalves^3*^, Pedro N.S. Rodrigues^1,2,3^

^1^ i3S - Instituto de Investigação e Inovação em Saúde, Universidade do Porto, Portugal

^2^ Iron and Innate Immunity, IBMC – Instituto de Biologia Molecular e Celular, Universidade do Porto, Portugal

^3^ ICBAS - Instituto de Ciências Biomédicas Abel Salazar, Universidade do Porto, Portugal

^4^ Programa Doutoral em Biologia Molecular e Celular (MCbiology), ICBAS - Instituto de Ciências Biomédicas Abel Salazar, Universidade do Porto, Portugal

*** Contact information for correspondence:**

João Vilares Neves

Iron and Innate Immunity

i3S - Instituto de Investigação e Inovação em Saúde, Rua Alfredo Allen 208, 4200-135 Porto, PORTUGAL

Tel: +351 226074900

E-mail: [jneves@ibmc.up.pt](mailto:jneves@ibmc.up.pt)

José Fernando Magalhães Gonçalves

ICBAS - Instituto de Ciências Biomédicas Abel Salazar, Rua Jorge Viterbo Ferreira 228, 4050-313 Porto, PORTUGAL

Tel: + 351 228428000

E-mail: [jfmg@icbas.up.pt](mailto:jfmg@icbas.up.pt)

**Keywords:** erythroferrone, hepcidin, teleost fish, iron overload, anemia, infection

**Supplementary Table 1.** Primers used for gene expression analysis.

|  | **Forward (5'🡪3')** | **Reverse (5'🡪3')** | **Primer Efficiency** |
| --- | --- | --- | --- |
| *actb* | CAGAAGGACAGCTACGT | GTCATCTTCTCCCTGTTGGC | 98,32% |
| *epo* | AGGCCAATCTGTGACCTGAG | GCAGTGCTGTGTTGGTGACT | 99,17% |
| *erfe* | GCGTTGAAGAACTGGAGAGC | TCGCACAGGAAACACACTCC | 98,73% |
| *hamp1* | CATTGCAGTTGCAGTGACACT | CAGCCCTTGTTGCCTCTG | 97,92% |
| *hamp2* | CTGCTGTCCCAGTCACTGA | ACCACATCCGCTCATATTAGG | 102,04% |
| *hbb* | CCAGGCTTTGACCAGACTTC | TGGACATCAGGGGTGAACTG | 100,2% |

**Supplementary Table 2.** Sequence identity, obtained using the SIAS server (http://imed.med.ucm.es/Tools/sias.html).

**Supplementary Figure 1 - Nucleotide and predicted amino acid sequence of sea bass erythroferrone**. Nucleotides are indicated above and numbered (outer columns). The deduced amino acid sequence is shown below the nucleotide sequence. Amino acids are indicated with italic letters and numbered (inner columns) ending with the TGA stop codon (represented by *). Predicted signal peptide cleavage site is dot underlined, N-glycosylation sites are represented in **bold**, a proline rich region is underlined and the C1q domain is thick underlined.
